# Feasibility of a Home‐Based Occupational Positioning Program for Children With Spastic Cerebral Palsy: A Preliminary Study

**DOI:** 10.1155/oti/2343887

**Published:** 2026-07-29

**Authors:** Meimanat Akbari, Malek Amini, Jamileh Abolghasemi, Akram Azad, Amirhossein Shafizadeh

**Affiliations:** ^1^ Department of Occupational Therapy, School of Rehabilitation Sciences, Iran University of Medical Sciences (IUMS), Tehran, Iran, iums.ac.ir; ^2^ Department of Biostatistics, School of Public Health, Iran University of Medical Sciences (IUMS), Tehran, Iran, iums.ac.ir; ^3^ Department of Occupational Therapy, Firoozgar Hospital, Iran University of Medical Sciences (IUMS), Tehran, Iran, iums.ac.ir

**Keywords:** cerebral palsy, feasibility study, functional skills, home-based intervention, occupation based practice, occupational therapy

## Abstract

**Purpose:**

The primary aim was to assess the feasibility of a home‐based occupational positioning program for children with spastic cerebral palsy (SCP), focusing on compliance, dosage, and satisfaction. A secondary aim was to explore its preliminary effectiveness on self‐care, mobility, social function, gross motor function, and goal attainment.

**Methods:**

This single‐group feasibility study, conducted prior to a larger RCT, included five children aged 3.5–6 years with SCP (GMFCS III–IV) without severe intellectual disability, and aimed at assessing feasibility while exploring preliminary effects. The home‐based occupational positioning program involved general and customized booklet‐based training, two in‐home sessions using Goal Attainment Scaling (GAS) to establish the home program, and 12 weekly remote sessions. Feasibility outcomes were assessed by compliance, dosage, and satisfaction, while preliminary effectiveness was evaluated using the PEDI (self‐care, mobility, and social function), Gross Motor Function Measure (GMFM‐88), and GAS.

**Results:**

All participants completed the study without adverse events or attrition. Mean compliance was 96.4*%* ± 4.93*%*, average satisfaction was 9.8 ± 0.44 on a 10‐point scale, and the average daily program time was 5.35 ± 3.11 h. Significant improvements (*p* < 0.05) were observed by Wilcoxon signed‐rank test in self‐care, mobility, and social function, as well as in GMFM‐88 and GAS scores.

**Conclusion:**

The home‐based occupational positioning program showed high feasibility, likely supported by family engagement, collaborative goal setting, real‐life implementation, caregiver education, and consistent remote support. Preliminary findings suggested potential benefits for self‐care, mobility, social function, gross motor function, and goal attainment.

**Trial Registration:**

Iranian Registry of Clinical Trials: IRCT20231012059697N1

## 1. Introduction

Spastic cerebral palsy (SCP) is the most prevalent subtype of cerebral palsy, accounting for approximately 80% of all diagnosed cases [1]. Core clinical features include persistent pathological reflexes, abnormal muscle tone, and impaired postural control, which interfere with postural development, disrupt movement patterns, reduce coordination, contribute to muscle weakness, impair balance, and lead to difficulties in maintaining functional positioning [2]. These impairments substantially limit children′s participation in self‐care, mobility, and social activities [3].

Various contemporary occupational therapy approaches are used in pediatric CP rehabilitation, including motor control and motor learning approaches, constraint‐induced movement therapy, and bimanual therapy [4], as well as emerging technologies such as virtual reality, robotics, functional electrical stimulation, and assistive devices [5]. Among these interventions, biomechanically based positioning strategies are commonly applied to improve postural alignment, enhance proximal stability, and support functional performance [6].

In the present study, occupational positioning refers to the integration of individualized positioning strategies within meaningful, family‐selected occupations that occur naturally in daily home routines. This approach combines biomechanical principles of postural alignment with occupation‐based and family‐centered practice, supported by a structured illustrated booklet and ongoing iterative therapist–family collaboration.

Unlike conventional positioning approaches that primarily focus on therapist‐directed postural alignment or equipment‐based strategies in clinical or structured home programs, the present intervention embeds individualized positioning within meaningful, family‐selected occupations performed in natural home environments. This represents a shift from positioning as an isolated biomechanical strategy toward positioning as an integrated component of occupation‐based performance. The intervention further incorporates individualized visual guidance, the use of readily available household materials, and ongoing remote therapist support to facilitate adaptation based on the child′s functional performance and family feedback.

Occupational positioning is conceptually grounded in occupation‐based practice, family‐centered practice, and occupational therapy home programs. From an occupation‐based perspective, it supports children′s participation in meaningful, goal‐directed occupations that reflect real‐life functional demands [7–9]. Consistent with family‐centered practice, positioning strategies are collaboratively developed with caregivers, emphasizing parent involvement, education, and shared decision‐making [10–12]. In alignment with occupational therapy home programs, these strategies are embedded within everyday routines in the home environment, promoting skill generalization and functional integration within natural contexts [13–15].

Previous studies have investigated positioning as a therapeutic strategy to support postural alignment and functional performance in children with cerebral palsy [16, 17]. However, these interventions have predominantly been delivered through therapist‐directed protocols, equipment‐based approaches, or structured home programs focusing primarily on postural control and functional task practice [18–21], without explicitly integrating individualized positioning strategies within meaningful, family‐selected occupations in natural home contexts. In addition, variability in implementation contexts and limited iterative adaptation within real‐life family routines may affect the transferability of these approaches to everyday occupational performance [20, 21].

However, occupation‐embedded positioning approaches within meaningful daily activities and collaborative family routines remain underexplored. Limited evidence exists regarding positioning strategies delivered through home‐based materials, individualized illustrated booklets, and structured therapist–family guidance for implementation in home contexts.

To address this gap, the present study developed and evaluated a home‐based occupational positioning program that embeds positioning within meaningful occupations, is codesigned with families, and is implemented within natural home routines. Prior to conducting a definitive randomized controlled trial (RCT), a feasibility study was conducted to assess the practicality and acceptability of the intervention in real‐world home settings. The primary aim of this study was to evaluate feasibility in children with SCP. Secondary objectives were to explore preliminary effects on functional skills—including self‐care, mobility, and social functioning (Pediatric Evaluation of Disability Inventory [PEDI]), goal attainment (Goal Attainment Scaling [GAS]), and gross motor function (Gross Motor Function Measure [GMFM])—as well as family experiences of participation in the program.

## 2. Methods

### 2.1. Study Design

This pilot study employed a single‐group, pretest‐posttest design to evaluate the feasibility of a home‐based occupational positioning program. Participants were recruited from rehabilitation centers in Tehran, Iran, between January and May 2024. Informed, voluntary consent was obtained from all caregivers prior to enrollment.

The study was structured as a feasibility trial to inform the implementation of a future large‐scale, repeated‐measures RCT. Ethical approval was obtained from the Research Ethics Committee of the Iran University of Medical Sciences (Ethics Code: IR.IUMS.REC.1402.589).

### 2.2. Study Participants

The inclusion criteria for parents were having basic literacy skills, being willing to participate in the study, and having sufficient time to implement the home‐based occupational positioning program. The exclusion criterion for parents was not being the child′s primary caregiver, as consistent implementation of the home‐based program required direct daily involvement. Postenrollment events—such as withdrawal of consent or failure to implement the program for more than 1 week—were considered dropouts.

The inclusion criteria for children were age between 1 and 7 years, a diagnosis of SCP confirmed by a pediatric neurologist, GMFCS levels II–IV [22], and absence of severe intellectual disability (IQ > 50 based on SPARCLE criteria [23]). The exclusion criteria included a history of orthopedic or reconstructive surgery within the past 6 months or botulinum toxin injection within the past 3 months. If a child underwent orthopedic surgery or received botulinum toxin injections during the intervention period, these cases were classified as dropouts.

### 2.3. Sample Size

This single‐arm feasibility trial, conducted prior to a definitive RCT, primarily is aimed at evaluating study processes rather than treatment effectiveness. As is common in feasibility research, no formal power calculation was performed. Instead, a small pragmatic sample (*n* = 5) was selected to assess study procedures while minimizing resource use. Predefined feasibility criteria (recruitment ≥ 60%, retention ≥ 80%, adherence ≥ 70%) were applied to evaluate the practicality and logistics of conducting a larger RCT. This approach aligns with recommendations that feasibility studies focus on estimating process parameters rather than testing hypotheses of effectiveness [24, 25].

However, this does not support conclusions regarding the efficacy of the home‐based occupational positioning program. In the subsequent full‐scale RCT, a sample of 42 participants per group (intervention and control) has been calculated to provide adequate power to detect the expected treatment effects.

### 2.4. Measures

#### 2.4.1. Feasibility Measures

Feasibility was evaluated based on compliance, dosage, and satisfaction. In addition, any adverse events or participant dropouts were documented. These measures were assessed by the therapist who delivered the intervention.

##### 2.4.1.1. Compliance

To assess compliance with the intervention, participants′ attendance was calculated by dividing the number of remote support sessions each caregiver completed by the total number of scheduled sessions and expressed as a percentage [26]. In this study, compliance was defined as the proportion of the 12 planned weekly sessions that were completed by each participant.

##### 2.4.1.2. Dosage

The dosage of the home‐based program was determined from weekly parental logbooks, in which caregivers recorded the duration of program implementation each day. For each participant, the mean daily duration (in hours) was calculated based on the 12 weekly records. Although no established reference exists for the optimal daily dosage of home‐based occupational positioning programs, this approach provides a practical and quantifiable measure of family engagement and supports the assessment of intervention feasibility [26, 27].

##### 2.4.1.3. Satisfaction

Parental satisfaction with the implementation of the home‐based program was measured using a 10‐point Likert‐type scale (1 = *completely dissatisfied*, 10 = *completely satisfied*). This single‐item measure provides sufficient response discrimination for subjective ratings [28]. During the final remote support session, caregivers were informed about the rating procedure and were asked to provide written feedback on their satisfaction level after the session.

#### 2.4.2. Outcome Measures

Outcome assessments included the PEDI, the GMFM, and the GAS, all administered before and after the intervention. A trained occupational therapist with 3 years of clinical experience in pediatric CP conducted all evaluations and was not involved in the intervention delivery. Outcome assessment was not blinded due to the single‐arm feasibility design; however, blinding will be implemented in the planned full‐scale RCT to minimize potential bias.

##### 2.4.2.1. PEDI [29]

The PEDI is a caregiver‐reported measure comprising three domains: functional skills (self‐care, mobility, and social function), caregiver assistance, and modifications. Items in the functional skills domain are scored as 0 (unable) or 1 (able), with higher scores reflecting greater independence. Domain scores are expressed as percentages of the maximum possible score. The Persian version of PEDI has demonstrated high reliability and internal consistency, with test‐retest reliability scores of 0.96–0.98 and Cronbach′s *α* values ranging from 0.94 to 0.98 [29]. The minimal clinically important difference (MCID) for the subdomains ranges from 6 to 15 points, with a mean of approximately 11% [30]. Only the functional skills subscales were used as the primary outcome measure for analysis in this study.

##### 2.4.2.2. GAS

GAS is a structured method for quantifying progress toward individualized therapeutic goals, using a five‐point scale ranging from +2 to −2 (xi). A score of 0 denotes the expected level of achievement; +1 and+2 represent greater‐than‐expected outcomes, whereas −1 and −2 reflect suboptimal or markedly limited progress, respectively. Each goal is rated for importance (0–3) and difficulty (0–3). A weighted value is calculated using the formula w_**i**_ = importance × difficulty. Preintervention and postintervention scores are applied to the overall GAS formula to determine change in goal attainment [31]. A change of ±10 points is generally considered the MCID for clinically meaningful improvement [32].

Overal GAS=50+10∑w1xi√0.70.3∑wi2+∑wi2.

##### 2.4.2.3. Gross Motor Function Measure‐88 (GMFM‐88) [33]

The GMFM‐88 is a standardized assessment tool for evaluating gross motor function in children aged 5 months to 16 years with motor impairments, including CP. It consists of five dimensions: lying and rolling (17 items), sitting (20 items), crawling and kneeling (14 items), standing (13 items), and walking, running, and jumping (24 items). Each item is rated on a 4‐point ordinal scale: 0 = *no initiation of movement*, 1 = *initiation with completion of ≤ 10% of the task*, 2 = *p* *a* *r* *t* *i* *a* *l* *c* *o* *m* *p* *l* *e* *t* *i* *o* *n* (10% to < 100%), 3 = *full completion of the task*. Domain scores are expressed as percentages of the maximum possible within each dimension, and the overall score is reported as the mean of all five domain percentages. The Persian version was validated by Salehi et al. showing excellent inter‐rater and intra‐rater reliability (ICC = 0.99) and high internal consistency (Cronbach^′^s *α* = 0.78–0.94) [33]. Reported MCID values for children with CP range from 0.1% to 3% [34].

### 2.5. Intervention

The intervention began with the development of a general educational booklet, Principles and Importance of Home‐Based Occupational Positioning, created by the study investigators to familiarize families with the principles and importance of occupational positioning in the home environment. Following this, an individualized home‐based occupational positioning program was designed for each child. The intervention was delivered by the first author, an occupational therapist and faculty member with more than 20 years of clinical experience working with children with cerebral palsy.

#### 2.5.1. Development of the General Educational Booklet

A 34‐page A4‐format educational booklet containing 134 illustrations was developed to enhance parental understanding of occupational positioning principles and to promote engagement and adherence to the intervention. The content was informed by existing evidence and theoretical frameworks, including the person–environment–occupation (PEO) model, occupation‐based practice, home‐based occupational therapy approaches, and biomechanical principles. The booklet covered the following sections: (1) definitions and classifications of CP, (2) muscle tone and reflexes, (3) common pathological reflexes and associated complications, (4) frequent postural deviations in children with SCP, (5) the role of home‐based positioning, (6) positioning during occupational engagement, (7) positioning principles across postures (supine, sitting, kneeling, and standing), and (8) low‐cost practical positioning strategies suitable for home settings.

The preliminary version underwent expert review by five pediatric occupational therapists and six occupational therapy faculty members with clinical experience in CP rehabilitation. Feedback obtained through written and verbal channels was used to revise the content and illustrations. Content validity was then evaluated using a content validity index (CVI) checklist assessing the clarity, relevance, and simplicity of the text and images. Twelve occupational therapy faculty members specializing in CP completed the evaluation. After revision of items with lower ratings, the final version achieved acceptable CVI scores across all components. In addition, five caregivers of children with SCP reviewed the finalized booklet and confirmed its clarity and usability.

Before initiating the intervention, families received the booklet and participated in a 1‐h in‐home training session. The session was delivered using a PowerPoint presentation and included lectures, discussion, and practical demonstrations to support caregiver understanding and preparedness for implementation.

#### 2.5.2. Home‐Based Occupational Positioning Program Design and Implementation

The intervention followed Novak′s five‐step model for home‐based occupational therapy. In Step 1, a therapeutic relationship was established between the therapist and the family. In Step 2, collaborative occupational goals were identified using the GAS. In Step 3, an individualized home‐based occupational positioning plan was developed to align with the child′s occupational needs and family routines. In Step 4, families implemented the program within daily routines and were supported through weekly remote sessions that provided ongoing guidance and monitored implementation. In Step 5, GAS was readministered to evaluate progress toward individualized goals [35].

The intervention included two in‐person home visits and 12 weekly remote follow‐up sessions, implemented as follows.

##### 2.5.2.1. Home Program—In‐Home Visit Sessions (Two Sessions)

During the first in‐home visit, the occupational therapist met with the child and family to establish a therapeutic relationship and assess the household context. The family received instruction based on the general educational booklet *Principles and Importance of Home-Based Occupational Positioning* (Step 1). Collaborative occupational goals—up to five—were then identified using GAS with the family, focusing on meaningful occupations embedded within daily routines (Step 2). Occupational goals were collaboratively selected by the therapist and family. An example of an occupation‐based goal developed using GAS is presented in Table 1. Goal selection was guided by two main principles: (1) the activity should be meaningful and frequently performed by the family, such as eating or play, and (2) the child should spend a considerable amount of time in the occupation in suboptimal positions, such as during television viewing or sleep. This approach ensured that the goals were individualized, contextually relevant, and targeted occupations in which positioning could enhance participation and engagement.

**Table 1:** Sample GAS goal (spoon feeding).

Level	Description
−2 (much less than expected)	Unable to maintain an appropriate posture for eating for at least 3 min but unable to use a spoon.
−1 (less than expected)	Able to maintain an appropriate position for at least 3 min but unable to use a spoon functionally.
0 (expected)	Able to maintain an appropriate position for 5 min and bring the spoon to the mouth at least five times with minimal assistance.
+1 (more than expected)	Brings the spoon to the mouth at least 10 times within 5 min, independently or with minimal assistance.
+2 (much more than expected)	Use of the affected hand for feeding in addition to grasping the spoon by other hand such as holding a plate or grasping a fork, independently or with minimal assistance.

*Note:* Goal: Within 3 months, the child will maintain an appropriate posture for eating and independently use a spoon for self‐feeding.

Abbreviation: GAS, Goal Attainment Scaling.

Individualized positioning strategies were then designed to support performance in the selected occupations, considering the child′s functional abilities, positioning needs, and available household resources. Photographs of each occupation were taken from multiple angles to ensure clarity and were used to develop a personalized booklet tailored to the child′s routine occupations. Each photograph was accompanied by key instructional elements, including the required level of physical assistance, suitable occupations for each position, essential safety precautions, and suggested durations for individual episodes of occupational performance. These suggested durations were based on the child′s abilities, comfort, and family context; however, no fixed overall daily dosage or prescribed intervention schedule was established. Caregivers were free to implement the positioning strategies flexibly within their daily routines. Before finalizing each positioning strategy, child and family comfort and safety were carefully evaluated (Step 3).

Representative images of occupational positioning are provided in Supplementary 1. In addition to standard assistive devices such as CP chairs, walkers, KAFOs, AFOs, and orthopedic shoes, household items were also adapted to support occupational positioning. Examples of these household‐based adaptations are presented in Supplementary 2

. The customized booklet was delivered during the second in‐home visit. The therapist provided hands‐on training using the booklet as a guide to ensure accurate implementation in the target occupations (Step 3). Families were also provided with a structured weekly logbook in a two‐dimensional matrix format (days of the week × occupational goals) and were instructed to record daily engagement times in the selected occupations. The schedule for weekly remote support sessions was established collaboratively with each family. Families were encouraged to facilitate the child′s participation in selected occupations within daily routines according to the customized booklet. Each home visit session lasted 2.5–3 h, and the interval between visits ranged from 1 to 2 weeks depending on family availability.

##### 2.5.2.2. Home Program—Remote Weekly Support Sessions (12 Sessions)

Throughout the 12‐week intervention, parents were instructed to capture and share photos and videos of the child during occupational engagement prior to each remote support session. Sessions were conducted via phone or online platforms and lasted 30–60 min, scheduled in coordination with each family. At the beginning of each session, parents reported on the implementation of the previous week. The therapist provided feedback to support appropriate implementation of the selected occupations and reinforced observed progress in the child′s performance. When challenges in implementing specific occupations were reported, targeted guidance and modifications were suggested as needed. If adjustments were required, detailed instructions were provided to support correct implementation.

The initial program was developed based on collaboratively selected occupational goals. During implementation, families also suggested additional positioning options to support occupational engagement. These suggestions were discussed during weekly sessions and, depending on clinical and contextual relevance, were either integrated, modified, or not implemented. This iterative process allowed the intervention to be continuously adapted in response to the child′s needs and family input.

Each week, one successfully implemented occupational positioning strategy was identified as the “Best Practice of the Week” and acknowledged accordingly. All families completed 12 remote support sessions (Step 4). In the final session, goal attainment was reassessed using GAS (Step 5). Concurrently, all children received at least two weekly sessions of conventional occupational therapy as part of their routine care. Participants′ experiences of the program were explored based on qualitative feedback collected from parents during weekly support sessions.

### 2.6. Data Analysis

Feasibility was evaluated by calculating participants′ mean compliance, satisfaction, and dosage values. Preintervention and postintervention change scores for PEDI, GMFM, and GAS were computed for each participant and compared against established MCID thresholds for each instrument. Due to the small sample size (*n* = 5), the Wilcoxon signed‐rank test assessed differences between preintervention and postintervention scores for all outcome measures. Statistical analyses were conducted using SPSS Version 27, with significance at *p* < 0.05.

## 3. Results

Six children were initially enrolled; however, one participant withdrew before initiating the intervention due to family‐related circumstances. The final sample included five children (two girls and three boys; mean age = 4.7 years) who completed the home‐based occupational positioning program with no adverse events or dropouts reported. Among them, two were diagnosed with spastic diplegia and three with spastic quadriplegia.

On average, participants attended 96.4% of the scheduled weekly remote support sessions. The mean daily program dosage was 5.35 h (2.45–10.37 h), and the average parental satisfaction score was 9.8 out of 10 (9–10). Table 2 presents detailed demographic and feasibility data, including age, sex, GMFCS level, CP type, compliance, dosage, and satisfaction. These results indicate high feasibility across all domains of implementation.

**Table 2:** Demographic data, compliance, dosage and satisfaction.

Participant	Age (year)	Sex	GMFCS	Type CP	Compliance (%)	Dosage (hour/day)	Satisfaction
1	6	Meal	4	Quadriplegic	100	5.28	10
2	3.5	Female	3	Diplegic	100	5.62	9
3	3.5	Female	4	Quadriplegic	100	10.37	10
4	4.5	Meal	4	Quadriplegic	91	2.99	10
5	5	Meal	3	Diplegic	91	2.54	10
Mean ± SD	4.5 ± 1.06	—	—	—	96.4 ± 4.93	5.35 ± 3.11	9.8 ± 0.44

Abbreviations: CP, cerebral palsy; GMFCS, Gross Motor Function Classification System.

**Figure 1:**
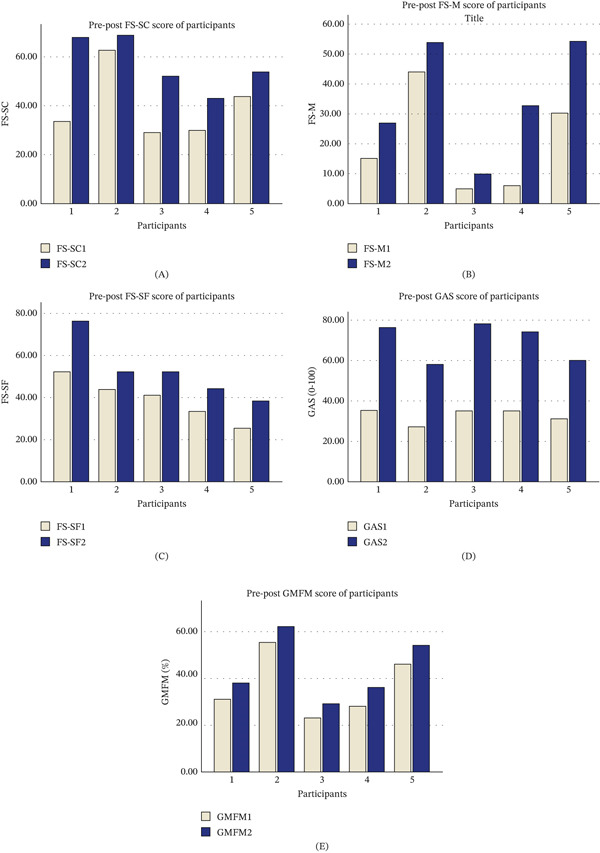
Graphs of before and after scores of 5 participants in outcome measures. Abbreviations: (A) FSS.SC_1_ = functional skill scale—self‐care before intervention, FSS.SC_2_ = functional skill scale‐self‐care after intervention. (B) FSS.M_1_ = functional skill scale—mobility before intervention, FSS.M_2_ = functional skill scale‐mobility after intervention. (C) FSS.SF_1_ = functional skill scale‐social function before intervention, FSS.SF_2_ = functional skill scale—social function after intervention (D) GAS_1_ = Goal Attainment Scaling before intervention, GAS_2_ = Goal Attainment Scaling after intervention, (E) GMFM_1_ = Gross Motor Function Measure‐88 before intervention, GMFM_2_ = Gross Motor Function Measure‐88 after intervention. Note: The horizontal axis displays the participant number (1–5) and the vertical axis displays the outcome measure scores. All participants′ scores showed improvement in all variables.

Individual‐level preintervention and postintervention scores and change scores are presented in Figure 1. Data are provided for the three domains of the PEDI functional skills subscale (self‐care, mobility, and social function), along with GAS and GMFM scores. All participants demonstrated improvements across all measured outcomes, with the largest effect observed in GAS scores.

Group‐level means and standard deviations for all outcome measures before and after the intervention, as well as the results of the Wilcoxon signed‐rank tests, are summarized in Table 3. In all cases, mean changes surpassed the respective MCID thresholds. Although the small sample size (*n* = 5) limits statistical power and generalizability, the Wilcoxon test revealed statistically significant improvements in all outcomes (*p* < 0.05). As illustrated in Table 3, each participant exhibited score increases across all outcome domains following the intervention.

**Table 3:** Descriptive statistics and Wilcoxon test results for outcome measures.

Participant	FSS‐SC			FSS‐M			FSS‐SF			GMFM			GAS		
	Before	After	Def	Before	After	Def	BEFOR	After	Def	Before	After	Def	Before	After	Def
1	34	68	34 ^∗^	15	27	12 ^∗^	52	76	24 ^∗^	21.2	38	6.8 ^∗^	36.1	76.1	41.2 ^∗^
2	63	69	9	44	54	10	44	52	8	55.6	62	6.4 ^∗^	27.5	58.7	31.^2^ ^∗^
3	29	52	13 ^∗^	5	10	5	41	52	11 ^∗^	23.4	29.8	6.4 ^∗^	35.1	78.2	43.1 ^∗^
4	30	43	13 ^∗^	6	33	27 ^∗^	33	44	11 ^∗^	28	36.6	8.6 ^∗^	35.6	74.9	39.3 ^∗^
5	44	54	10	30	545	24 ^∗^	26	38	12 ^∗^	45	54.6	8.4 ^∗^	31.3	60.2	28.9 ^∗^
Mean (SD)	40 (14.16)	57.7 (11.12)	17.7 ^∗^ (11.30	20 (16.75)	35.6 (18.80)	15.6 ^∗^ (9.45)	39.2 (10.03)	52.4 (11.12)	13.2 ^∗^ (6.22)	36.88 (13.5)	44.2 (13.49)	7.32 ^∗^ (1.09)	32.92 (3.49)	69.66 (9.41)	36.74 ^∗^ (6.31)
Wilcoxon test	*Z* = 2.023			*Z* = 2.023			*Z* = 2.023			*Z* = 2.032			*Z* = 2.023		
	Sig = 0.043			Sig = 0.043			Sig = 0.043			Sig = 0.042			Sig = 0.043		

*Note:* Items with (∗) indicate increases greater than the MCID for each participant and mean change. Comparing the before and after means in all variables with the Wilcoxon test showed the significant increase.

Abbreviations: Def, deference (after‐before); FSS.M, functional skill scale—mobility; FSS.SC, functional skill scale—social functional; FSS.SF, functional skill scale—self‐care; GAS, Goal Attainment Scaling; GMFM, Gross Motor Function Measure‐88; MCID, minimal clinically important difference; SD, standard deviation.

### 3.1. Family Experience of the Home‐Based Occupational Positioning Program

Families reported several positive experiences that contributed to the program′s high feasibility and parental engagement. Participation in goal‐setting and the opportunity for families to personalize the title of the customized booklet fostered a sense of ownership of the program. The general educational booklet enhanced parental awareness of maladaptive positions—such as W‐sitting—and their associated complications, leading to greater attention to inappropriate positioning during daily occupations. Including photographs of the child within the personalized booklet further increased parental satisfaction and supported the child′s engagement and participation in the program.

Another positive experience reported by families was the effective use of existing resources in the home, school, and car environments to support occupational performance without requiring specialized or costly devices. This adaptive use of household resources, together with therapist support through photo and video review, facilitated ongoing implementation of the program between therapist contacts. Moreover, the program′s emphasis on meaningful occupations alongside proper positioning enabled families to make informed adjustments based on the child′s functional progress. For example, some parents progressed an occupation—such as drawing in kneeling—from tabletop activities to wall‐supported positioning, reflecting an increased understanding of the child′s abilities and participation potential.

The structured use of daily logbooks and weekly remote support sessions further improved family engagement. These sessions provided an emotionally supportive environment where families could share parenting challenges and receive empathy. Observable progress, including increased duration of task engagement, higher frequency of occupational actions (e.g., spoon‐to‐mouth repetitions), and improved trunk and limb alignment, served as powerful motivators for continued participation. Over time, these repeated occupational experiences in optimized positions enhanced children′s self‐efficacy and confidence in selected occupations.

Despite these positive experiences, families also reported several challenges. Initially, caregivers described difficulty maintaining consistent implementation, particularly because proper occupational positioning required frequent reminders and ongoing attention. However, these challenges gradually decreased over time as caregivers became more proficient in implementing positioning strategies during occupations and children demonstrated greater independence in performing targeted activities. In some cases, the use of multiple household items for occupational positioning temporarily contributed to environmental disorganization within the home. Nevertheless, this challenge also diminished gradually as families became more familiar and efficient in implementing the positioning strategies.

## 4. Discussion

### 4.1. Feasibility

This single‐arm feasibility study primarily aimed to assess the practical aspects of implementing a 12‐week home‐based occupational positioning program for children with SCP, and results demonstrated its high feasibility. The intervention achieved excellent compliance, dosage, and parental satisfaction outcomes, potentially reflecting the integration of individualized positioning strategies within meaningful family‐selected occupations and natural home routines. No adverse events or dropouts were reported throughout the intervention period.

A compliance rate exceeding 95% was observed, which falls at the higher end of rates reported in comparable home‐based rehabilitation programs (56%–99%) [26]. This high adherence may have been facilitated by active family involvement and the integration of positioning strategies into meaningful daily routines and family‐selected occupations, factors that have been associated with improved engagement and compliance in home‐based interventions [15].

Another key factor was the flexibility of the home‐based model. Families could determine the timing and frequency of intervention activities according to their child′s natural rhythms, daily routines, and family‐selected occupations. This contrasts with protocols, which often follow fixed repetitions and rigid sequences [16, 36]. Such autonomy may enhance compliance by enabling parents to engage the child during periods of optimal attention and participation while adapting interventions to meaningful real‐life contexts [14, 15].

Educational strategies also supported adherence. Families received a general booklet on positioning principles, a personalized manual illustrating the child in meaningful occupational contexts, and ongoing remote coaching. Gmmash et al. highlighted that informed parents are more likely to comply with home programs [37]. Moreover, coaching enhances parental understanding of treatment principles and is essential for maintaining engagement in home‐based, occupation‐integrated interventions [26, 38].

A central element of the intervention was collaborative goal‐setting using GAS, where the therapist and family jointly identified meaningful occupations. This process enhanced family investment and facilitated implementation. Previous studies have highlighted collaborative design as a key strategy for empowering caregivers, reducing emotional distress, and ensuring goals align with family values. When goals match family priorities, they act as motivational facilitators, supporting both compliance and satisfaction [15, 39, 40].

Perceived progress in the child, particularly when reinforced by therapist feedback during remote sessions, strongly motivated continued engagement. These findings align with Verhaegh et al., who reported that visible improvements and positive feedback enhance caregiver motivation and persistence [41], and with Ortega‐Martínez et al., who emphasized the motivational role of encouragement in family‐centered interventions [14]. In this study, engagement in meaningful daily occupations, such as eating and play—prioritized by all five families—provided highly motivating contexts for both children and caregivers, promoting greater participation.

The intervention yielded an average daily dosage of 5.35 h. A recent systematic review of home‐based interventions for children with cerebral palsy reported a wide range of intervention dosage, from 70 min to 56 h per week; although functional improvement typically requires 30–40 h of weekly engagement, task‐specific skill acquisition may be achieved with lower intensities [26]. With a mean weekly dosage of approximately 37 h, the present study aligns with these thresholds. The flexible, family‐adapted schedule—allowing caregivers to determine when and how to implement positioning within daily routines—likely facilitated consistent practice and may have contributed to observed improvements in PEDI and GMFM outcomes.

A likely reason for the relatively high dosage is the integration of positioning strategies into frequently performed, meaningful, family‐selected occupations. Embedding the intervention within daily routines may have reinforced commitment and facilitated repetition. In contrast, other studies reported shorter implementation durations. For example, Medeiros et al. described a collaboratively designed intervention averaging 1.34 h per day, focusing on rolling, creeping, and standing through play without integration into daily routines [42]. Similarly, Palomo‐Carrión et al. implemented a 10‐week modified constraint‐induced movement therapy combined with bimanual intensive training protocol totaling 94 h (averaging 1.34 h per day) [27], and Ortega‐Martínez et al. used mirror therapy five times per week for only 30 min per session [14]. In the current study, selecting high‐frequency occupations such as eating and play—and, in two families, sleep‐related positioning—allowed repeated practice of positioning strategies, contributing to the higher overall dosage.

Parental satisfaction was high, with a mean score of 9.8/10, reflecting strong engagement and perceived effectiveness of the home‐based, family‐centered occupational positioning program. This aligns with reports by Palomo‐Carrión et al. and Choi et al., who also found high satisfaction with home‐based interventions [15, 43]. A systematic review identified ease of implementation, visible child progress, and caregiver empowerment as key factors influencing satisfaction, which were similarly reflected in parent‐reported benefits in the present study [26].

Overall, the findings indicate that the home‐based occupational positioning program is highly feasible for children with SCP and their families. High adherence, strong parental satisfaction, and the absence of adverse events or dropouts suggest that the intervention was both practical and acceptable in real‐world home contexts. These outcomes appear to be supported by the integration of positioning strategies within meaningful, family‐selected occupations, flexible delivery within daily routines, collaborative goal‐setting, and ongoing therapist–family engagement, highlighting the potential of occupation‐based, family‐centered positioning approaches for successful home implementation and supporting further evaluation in larger controlled trials.

### 4.2. Preliminary Effectiveness

In addition to feasibility, preliminary exploratory analyses were conducted to provide initial insights into the potential effects of the home‐based, family‐centered occupational positioning program on self‐care, mobility, social function, gross motor function, and goal attainment. Given the small sample size and single‐group design, these results should be interpreted with caution and cannot be considered conclusive evidence of effectiveness. Nevertheless, the observed changes may reflect key features of the intervention, including the integration of positioning strategies within meaningful, family‐selected daily occupations, collaborative goal‐setting with caregivers, and flexible scheduling tailored to each child’s natural routines.

Regarding outcomes, observed gains in PEDI domains—self‐care, mobility, and social function—may stem from integrating individualized positioning strategies into meaningful, family‐selected daily activities, along with gradual task mastery, consistent family support, and positive reinforcement. These features likely enhanced children′s engagement and participation, facilitating functional improvements. These findings are consistent with Ko et al., who reported functional gains following a task‐oriented, home‐based activity support program [44].

By contrast, Sel et al. reported improvements in self‐care and mobility but not in social function following a 12‐week telerehabilitation program that primarily emphasized physical activities such as assisted walking and mobility training in a daycare setting [45]. Unlike these interventions, the present home‐based program specifically targeted interpersonally embedded, meaningful daily occupations (e.g., mealtime with family and peer play), thereby supporting both physical and psychosocial functioning. The observed improvements in social function may also be related to enhanced eye contact and visual tracking, increased respiratory efficiency, and improved verbal interaction, all facilitated by optimized head and trunk stability achieved through individualized positioning strategies. Similarly, Choi et al. reported improvements in self‐care but no significant changes in mobility or social function after a 6‐week virtual reality program [43]; these limitations may be attributed to the shorter intervention duration and the primary focus on distal upper‐limb motor practice, which may have been insufficient to influence mobility or social outcomes.

A key factor in goal attainment, as measured by GAS, was the families′ strong commitment to collaboratively set goals with the therapist. For example, several parents reported that prior to the program they had never offered a spoon to their child at mealtimes. During the intervention, small but meaningful steps—such as grasping, holding, or moving the spoon toward the mouth—were important milestones. These achievements were supported by individualized positioning of the plate, trunk, and upper limbs, allowing children to participate in self‐care even before fully independent feeding. This aligns with Palomo‐Carrión et al., who reported increases of 30 points or more in GAS scores following a home early‐intervention program combining modified constraint‐induced movement therapy and bimanual intensive training [15]. These findings highlight the value of embedding GAS in home programs with collaborative goal‐setting and family involvement to enhance goal attainment.

In the present study, occupational performance was facilitated in various optimal positions—including prone, sitting (most frequently used), kneeling, and standing—selected according to activity demands and each child′s motor abilities. These individualized positioning strategies may have contributed to improvements in GMFM scores by reducing abnormal reflexes and spasticity, while enhancing proximal stability and postural control. Embedding these positions within meaningful daily activities allowed children to repeatedly practice functional movements in natural contexts, thereby reinforcing skill acquisition. Supporting this, Rauf et al. demonstrated that therapeutic positioning—including optimized sitting, nighttime alignment, and use of standing frames—significantly enhanced GMFM scores in children with spastic quadriplegia [19].

Not all studies have reported similar motor gains. For example, Goswami et al. found that a family‐centered, activity‐based intervention, although feasible and well‐tolerated, did not significantly improve GMFM scores over 6 months [46]. This discrepancy may reflect differences in intervention design: unlike the present study, which focused on family‐identified, meaningful occupational goals and embedded positioning strategies within daily routines, Goswami′s program used standardized, therapist‐prescribed activities applied uniformly. The lack of personalized, occupation‐based tasks and fixed therapist‐determined dosage may have limited gross motor improvements.

To our knowledge, no previous study has used a structured, child‐specific illustrated booklet to guide individualized positioning during meaningful occupations embedded in real‐life family routines. Although previous research has highlighted the usefulness of general educational booklets for parents and caregivers of children with cerebral palsy—for example, Lima et al. provided a booklet containing intervention guidelines, step‐by‐step activities, and practical examples to support home‐based program [47]—these booklets did not include individualized photographs of the child, nor were the suggested activities embedded within meaningful daily occupations or family routines. This approach demonstrates how individualized visual guidance can facilitate meaningful, family‐selected occupations in home settings.

Overall, these exploratory findings suggest that the home‐based, family‐centered occupational positioning program—embedding individualized positioning strategies within meaningful daily occupations and emphasizing collaborative goal‐setting—may have positive effects on self‐care, mobility, social function, gross motor function, and goal attainment. Given the exploratory nature of this feasibility study, these findings should be interpreted cautiously and primarily serve to guide outcome selection and study design in future trials.

## 5. Study Limitations and Future Directions

This study utilized a single‐group prepost design without a control group, limiting the generalizability of the findings and precluding causal inference. The small sample size further reduced statistical power. In addition, all participants continued receiving conventional occupational therapy throughout the study. Therefore, the observed functional improvements cannot be attributed solely to the home‐based occupational positioning program. A definitive randomized controlled trial (RCT), currently underway, is expected to provide more robust evidence regarding the unique contribution of this intervention.

The absence of long‐term follow‐up also limits conclusions regarding the sustainability, amplification, or attenuation of observed improvements over time. Furthermore, intervention fidelity was not formally assessed in this pilot study. Although weekly online sessions with the therapist and ongoing family feedback were used to support consistent implementation, fidelity will be formally evaluated in the subsequent RCT.

Finally, although the intervention was family‐centered, family‐related outcomes such as parental confidence, competence, and stress were not formally measured, despite some of these aspects being indirectly reflected in the families′ experiences. Future studies should include these outcomes to provide a more comprehensive understanding of the intervention′s impact on both children and their families.

## 6. Conclusion

A 12‐week home‐based occupational positioning program was found to be a feasible and well‐tolerated intervention for families of children with SCP, with high compliance, substantial intervention dosage, strong caregiver satisfaction, and no reported adverse events or dropouts. Key features supporting feasibility included active family involvement, flexible integration into daily routines, individualized goal‐setting, structured educational support, remote guidance, and ongoing motivational reinforcement.

Preliminary findings suggest that embedding individualized positioning strategies within meaningful daily occupations may support improvements in self‐care, mobility, social function, goal attainment, and gross motor performance in children with SCP. However, given the feasibility design and small sample size, these findings should be interpreted cautiously. Larger controlled studies are needed to confirm effectiveness and determine the long‐term impact of the intervention on both child and family outcomes.

## Funding

No funding was received for this manuscript.

## Conflicts of Interest

The authors declare no conflicts of interest.

## Supporting information


**Supporting Information** Additional supporting information can be found online in the Supporting Information section. Supporting File 1 includes images from the five participants′ customized booklet, indicating the selected occupations in suggested positions and GMFCS level of each participant. Supporting File 2 indicates examples of household items used for occupational positioning, which includes the most common items such as sofa cushions, pillows, adult dining chair, and more in various applications for participants, and tips and cautions associated with them.

## Data Availability

Research data are not publicly shared.

## References

[1] Patel D. R. , Neelakantan M. , Pandher P. , and Merrick J. , Cerebral Palsy in Children: A Clinical Overview, Translational Pediatrics. (2020) 9, no. Supplement 1, S125–S135, 10.21037/tp.2020.01.01, 32206590. 32206590 PMC7082248

[2] Panteliadis C. P. , Hagel C. , Karch D. , and Heinemann K. , Cerebral Palsy: A Lifelong Challenge Asks for Early Intervention, Open Neurology Journal. (2015) 9, no. 1, 45–52, 10.2174/1874205X01509010045, 26191093. 26191093 PMC4503828

[3] Phipps S. and Roberts P. , Predicting the Effects of Cerebral Palsy Severity on Self-Care, Mobility, and Social Function, American Journal of Occupational Therapy. (2012) 66, no. 4, 422–429, 10.5014/ajot.2012.003921, 22742690. 22742690

[4] O′Brien J. C. and Kuhaneck H. , Case-Smith′s Occupational Therapy for Children and Adolescents, 2019, Elsevier Health Sciences.

[5] Martínez-Rodríguez L. , Sánchez-López A. M. , Rodríguez-Martín M. M. , Gómez-Pérez I. , and Torres-Martín J. , New Technological Approaches in Occupational Therapy for Pediatric Cerebral Palsy: A Systematic Review, Healthcare. (2025) 13, no. 5, 10.3390/healthcare13050459, 40077021. PMC11899563 40077021

[6] Kramer P. , Frames of Reference for Pediatric Occupational Therapy, 2018, Lippincott Williams & Wilkins.

[7] Che Daud A. Z. , Yau M. K. , Barnett F. , and Judd J. , Occupation-Based Intervention in Hand Injury Rehabilitation: Experiences of Occupational Therapists in Malaysia, Scandinavian Journal of Occupational Therapy. (2016) 23, no. 1, 57–66, 10.3109/11038128.2015.1062047, 26153367. 26153367

[8] Estes J. and Pierce D. E. , Pediatric Therapists′ Perspectives on Occupation-Based Practice, Scandinavian Journal of Occupational Therapy. (2012) 19, no. 1, 17–25, 10.3109/11038128.2010.547598, 21198338. 21198338

[9] Laverdure P. and Beisbier S. , Occupation- and Activity-Based Interventions to Improve Performance of Activities of Daily Living, Play, and Leisure for Children and Youth Ages 5 to 21: A Systematic Review, American Journal of Occupational Therapy. (2021) 75, no. 1, 7501205050p1–7501205050p24, 10.5014/ajot.2021.039560, 33399053. 33399053

[10] Lage C. R. , Wright S. , de Souza Monteiro R. G. , and Boshoff K. , Collaborative Practices With Parents and Primary Caregivers in Pediatric Occupational Therapy: A Scoping Review Protocol, JBI Evidence Synthesis. (2022) 20, no. 6, 1593–1600, 10.11124/JBIES-21-00142, 35124686. 35124686

[11] Agarwal S. , Scher M. S. , and Tilton A. , Cerebral Palsy and Rehabilitative Care: the Role of Home-Based Care and Family-Centered Approach, Indian Pediatrics. (2021) 58, no. 9, 813–814, 10.1007/s13312-021-2298-z, 34508333. 34508333 PMC8464190

[12] Palomo-Carrión R. , Romay-Barrero H. , Pinero-Pinto E. , Romero-Galisteo R.-P. , López-Muñoz P. , and Martínez-Galán I. , Early Intervention in Unilateral Cerebral Palsy: Let’s Listen to the Families! What Are Their Desires and Perspectives? A Preliminary Family-Researcher Co-Design Study, Children. (2021) 8, no. 9, 10.3390/children8090750, 34572182. PMC8467316 34572182

[13] Ferre C. L. , Brandão M. B. , Hung Y.-C. , Carmel J. B. , and Gordon A. M. , Feasibility of Caregiver-Directed Home-Based Hand-Arm Bimanual Intensive Training: a Brief Report, Developmental Neurorehabilitation. (2015) 18, no. 1, 69–74, 10.3109/17518423.2014.948641, 25180530. 25180530 PMC4511850

[14] Ortega-Martínez A. , Sánchez J. L. , García M. , and Livanelioglu A. , Feasibility of a Home-Based Mirror Therapy Program in Children With Unilateral Spastic Cerebral Palsy, Healthcare. (2023) 11, no. 12, 10.3390/healthcare11121797, 37372915. PMC10298458 37372915

[15] Palomo-Carrión R. , Romay-Barrero H. , Pinero-Pinto E. , Romero-Galisteo R. P. , Coello-Villalón M. , Ferri-Morales A. , López-Muñoz P. , and Lirio-Romero C. , Feasibility of Home-Based Early Infant Hybrid Therapy in Children With Unilateral Cerebral Palsy, Journal of Clinical Medicine. (2024) 13, no. 22, 10.3390/jcm13226725, 39597871. PMC11594390 39597871

[16] Wadee A. N. , El-DIEN S. S. S. , and Elshinnawy A. M. , Influence of prone Positioning on Gross Motor Development in Children With Spastic Diplegic Cerebral Palsy, Journal of Advanced Pharmacy Education & Research. (2020) 10, no. 3.

[17] Sahinoğlu D. , Coşkun G. , and Bek N. , Effects of Different Seating Equipment on Postural Control and Upper Extremity Function in Children With Cerebral Palsy, Prosthetics and Orthotics International. (2017) 41, no. 1, 85–94, 10.1177/0309364616637490, 27025243. 27025243

[18] Mahmood Q. , Habibullah S. , and Aurakzai H. U. , Effectiveness of Simple and Basic Home-Based Exercise Programs Including Pediatric Massage Executed by Caregivers at Their Homes in the Management of Children With Spastic Cerebral Palsy: A Randomized Controlled Trial, Journal of Pediatric Rehabilitation Medicine. (2024) 17, no. 1, 97–106, 10.3233/PRM-220135, 38427509. 38427509 PMC10977413

[19] Rauf W. , Sarmad S. , Khan I. , and Jawad M. , Effect of Position on Gross Motor Function and Spasticity in Spastic Cerebral Palsy Children, Journal of the Pakistan Medical Association. (2021) 71, no. 3, 801–805, 10.47391/JPMA.1213. 34057924

[20] Hamzah F. and Ramli S. H. , A Systematic Review of Assistive Technology Devices to Promote Independent Living in Children With Cerebral Palsy, Proceedings of the 2nd International Conference on Design Industries and Creative Culture, DESIGN DECODED 2021, 2022, European Alliance for Innovation, 10.4108/eai.24-8-2021.2315270.

[21] Cheng H.-Y. K. , Lin C.-Y. , and Chen Y.-T. , Exploring Growth-Stage Variations in Home Use of Positioning and Mobility Assistive Technology for Children With GMFCS IV cerebral palsy: Parental Insights and Challenges, Bioengineering. (2025) 12, no. 3, 10.3390/bioengineering12030241, 40150705. PMC11939245 40150705

[22] Riahi A. , Rassafiani M. , AkbarFahimi N. , Sahaf R. , and Yazdani F. , The Cross-Cultural Validation and Test-Retest and Inter-Rater Reliability of the Persian Translation of parent version of the Gross Motor Function Classification System for Children With Cerebral Palsy, Archives of Rehabilitation / Journal of Rehabilitation. (2013) 13, no. 5, 25–30.

[23] Colver A. and SPARCLE Group , Study Protocol: SPARCLE—A Multi-Centre European Study of the Relationship of Environment to Participation and Quality of Life in Children With Cerebral Palsy, BMC Public Health. (2006) 6, no. 1, 10.1186/1471-2458-6-105, 16638126. PMC1459857 16638126

[24] Eldridge S. M. , Chan C. L. , Campbell M. J. , Bond C. M. , Hopewell S. , Thabane L. , Lancaster G. A. , and PAFS Consensus Group , CONSORT 2010 Statement: Extension to Randomised Pilot and Feasibility Trials, British Medical Journal. (2016) 355, i5239, 10.1136/bmj.i5239, 27777223. 27777223 PMC5076380

[25] Billingham S. A. , Whitehead A. L. , and Julious S. A. , An Audit of Sample Sizes for Pilot and Feasibility Trials Being Undertaken in the United Kingdom Registered in the United Kingdom Clinical Research Network database, BMC Medical Research Methodology. (2013) 13, no. 1, 10.1186/1471-2288-13-104, 23961782. PMC3765378 23961782

[26] Beckers L. W. , Geijen A. J. , Kleijnen M. M. , A A Rameckers E. , L A P Schnackers M. , J E M Smeets R. , and Janssen-Potten Y. J. M. , Feasibility and effectiveness of Home-Based Therapy Programmes for Children With Cerebral Palsy: A Systematic Review, BMJ Open. (2020) 10, no. 10, e035454, 10.1136/bmjopen-2019-035454, 33028544. PMC7539606 33028544

[27] Palomo-Carrión R. , Romay-Barrero H. , Lirio-Romero C. , Arroyo-Fernádez R. , M-Guijarro-Herraiz M. , and Ferri-Morales A. , Feasibility of Family-Directed Home-Based Bimanual Intensive Therapy Combined With Modified Constraint Induced Movement Therapy (h-BITmCI) in Very Low and Low Bimanual Functional Level: A Brief Report, Developmental Neurorehabilitation. (2023) 26, no. 1, 63–70, 10.1080/17518423.2022.2099993, 35833864. 35833864

[28] Dawes J. , Do Data Characteristics Change According to the Number of Scale Points Used? An Experiment Using 5-Point, 7-Point and 10-Point Scales, International Journal of Market Research. (2008) 50, no. 1, 61–104, 10.1177/147078530805000106.

[29] Moradi Abbasabadi M. , Akbarfahimi N. , Hosseini S. A. , and Rezasoltani P. , Reliability of the Persian Version of the Pediatric Evaluation of Disability Inventory in 3 to 9-Year Old Children With Cerebral Palsy, Journal of Mazandaran University of Medical Sciences. (2015) 25, no. 130, 129–137.

[30] Iyer L. V. , Haley J. A. , Watkins S. , and Jensen M. P. , Establishing Minimal Clinically Important Differences for Scores on the Pediatric Evaluation of Disability Inventory for Inpatient Rehabilitation, Physical Therapy. (2003) 83, no. 10, 888–898, 10.1093/ptj/83.10.888, 14519060. 14519060

[31] Steenbeek D. , Ketelaar M. , Galama K. , and Gorter J. W. , Goal Attainment Scaling in Paediatric Rehabilitation: A Critical Review of the Literature, Developmental Medicine & Child Neurology. (2007) 49, no. 7, 550–556, 10.1111/j.1469-8749.2007.00550.x, 17593130. 17593130

[32] Tennant A. , Goal Attainment Scaling: Current Methodological Challenges, Disability and Rehabilitation. (2007) 29, no. 20–21, 1583–1588, 10.1080/09638280701618828, 17882728. 17882728

[33] Salehi R. , Keshavarz A. , Negahban H. , Saeedi A. , Shiravi A. , Ghorbani S. , Taghizade G. , and Azizi R. , Development of the Persian Version of Gross Motor Function Measure-88 (GMFM-88): A Study of Reliability, Trends in Medical Research. (2015) 10, no. 3, 69–74, 10.3923/tmr.2015.69.74.

[34] Storm F. A. , Heller B. , Datar R. , Petrarca M. , and Castelli E. , Minimum Clinically Important Difference of Gross Motor Function and Gait Endurance in Children With Motor Impairment: a Comparison of Distribution-Based Approaches, BioMed Research International. (2020) 2020, 6903045, 10.1155/2020/2794036, 32509855. PMC7246400 32509855

[35] Novak I. and Cusick A. , Home Programmes in Paediatric Occupational Therapy for Children With Cerebral Palsy: Where to Start?, Australian Occupational Therapy Journal. (2006) 53, no. 4, 251–264, 10.1111/j.1440-1630.2006.00577.x.

[36] Kara O. K. , Yardimci B. N. , Sahin S. , Orhan C. , Livanelioglu A. , and Soylu A. R. , Combined Effects of Mirror Therapy and Exercises on the Upper Extremities in Children With Unilateral Cerebral Palsy: A Randomized Controlled Trial, Developmental Neurorehabilitation. (2020) 23, no. 4, 253–264, 10.1080/17518423.2019.1662853, 31514564. 31514564

[37] Gmmash A. S. , Effgen S. K. , Skubik-Peplaski C. , and Lane J. D. , Parental Adherence to Home Activities in Early Intervention for Young Children With Delayed Motor Development, Physical Therapy. (2021) 101, no. 4, pzab023, 10.1093/ptj/pzab023, 33481991. 33481991

[38] Svensson K. , Andersson M. , Karlsson P. , and Johansson L. , Parents in the Driver′s Seat—Experiences of Parent-Delivered Baby-mCIMT Coached Remotely, Journal of Clinical Medicine. (2024) 13, no. 16, 10.3390/jcm13164864, 39201006. PMC11355282 39201006

[39] Löwing K. , Hamer E. G. , Bexelius A. , and Brogren Carlberg E. , Exploring the Relationship of Family Goals and Scores on Standardized Measures in Children With Cerebral Palsy, Using the ICF-CY, Developmental Neurorehabilitation. (2011) 14, no. 2, 79–86, 10.3109/17518423.2011.552088, 21410399. 21410399

[40] Harniess P. A. , Gibbs D. , Bezemer J. , and Basu A. P. , Parental Engagement in Early Intervention for Infants With Cerebral Palsy—A Realist Synthesis, Child: Care, Health and Development. (2022) 48, no. 3, 359–377, 10.1111/cch.12916, 34622968. 34622968 PMC9298289

[41] Verhaegh A. P. M. , Nuijen N. B. , Aarts P. B. M. , Nijhuis-van der Sanden M. , Willemsen M. A. , Groen B. E. , and Vriezekolk J. E. , Parents′ Experiences With a Home-Based Upper Limb Training Program Using a Video Coaching Approach for Infants and Toddlers With Unilateral Cerebral Palsy: A Qualitative Interview Study, BMC Pediatrics. (2022) 22, no. 1, 10.1186/s12887-022-03432-w, 35768858. PMC9245237 35768858

[42] de Medeiros N. L. , Ferreira F. R. , Vaz D. V. , Silva H. A. , An M. , Palisano R. J. , Leite H. R. , and Camargos A. C. R. , Family-Professional Collaborative Physical Therapy Intervention via Telehealth for Children With Developmental Disabilities: A Mixed-Method Feasibility Study, Physical & Occupational Therapy In Pediatrics. (2025) 45, no. 3, 375–388, 10.1080/01942638.2024.2447024, 39757349. 39757349

[43] Choi J. Y. , Yi S.-H. , Shim D. , Yoo B. , Park E. S. , and Rha D. , Home-Based Virtual Reality-Enhanced Upper Limb Training System in Children With Brain Injury: A Randomized Controlled Trial, Frontiers in Pediatrics. (2023) 11, 1131573, 10.3389/fped.2023.1131573, 37274815. 37274815 PMC10233002

[44] Ko S.-H. , Kim J.-E. , and Koo J.-W. , Effect of Home Activity Support Program on Daily Living Performance Skills in Children With Cerebral Palsy and Their Parents, Physical Therapy Rehabilitation Science. (2022) 11, no. 1, 24–31, 10.14474/ptrs.2022.11.1.24.

[45] Sel S. A. , Günel M. K. , Erdem S. , and Tunçdemir M. , Effects of Telerehabilitation-Based Structured Home Program on Activity, Participation and Goal Achievement in Preschool Children With Cerebral Palsy: A Triple-Blinded Randomized Controlled Trial, Children. (2023) 10, no. 3, 10.3390/children10030424, 36979982. PMC10047243 36979982

[46] Goswami J. N. , Sankhyan N. , and Singhi P. , Add-on Home-Centered Activity-Based Therapy vs Conventional Physiotherapy in Improving Walking Ability at 6-Months in Children With Diplegic Cerebral Palsy: A Randomized Controlled Trial, Indian Pediatrics. (2021) 58, no. 9, 826–832, 10.1007/s13312-021-2301-8, 34047715. 34047715

[47] Lima C. R. G. , de Abreu R. W. F. , Verdério B. N. , Brugnaro B. H. , Santos M. M. , dos Santos A. N. , Morgan C. , and Rocha N. A. C. F. , Early Intervention Involving Specific Task-Environment-Participation (STEP) Protocol for Infants at Risk: A Feasibility Study, Physical & Occupational Therapy In Pediatrics. (2023) 43, no. 3, 303–320, 10.1080/01942638.2022.2142084, 36329671. 36329671

