# Supplementary material for: Feasibility of a Home‐Based Occupational Positioning Program for Children With Spastic Cerebral Palsy: A Preliminary Study
Source: Occup Ther Int. 2026 Jul 29;2026:2343887. doi: 10.1155/oti/2343887 (PMC13420267; doi:10.1155/oti/2343887)
Supplement: Supplementary file 1 — Supporting Information Additional supporting information can be found online in the Supporting Information section. Supporting File 1 includes images from the five participants′ customized booklet, indicating the selected occupations in suggested positions and GMFCS level of each participant. Supporting File 2 indicates examples of household items used for occupational positioning, which includes the most common items such as sofa cushions, pillows, adult dining chair, and more in various applications for participants, and tips and cautions associated with them. [file OTI-2026-2343887-s001.docx]

**SUPPLEMENTARY 1:** Representative images of occupational positioning


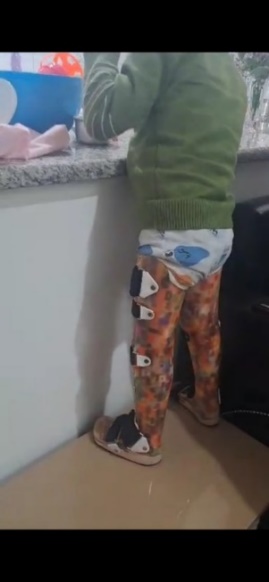

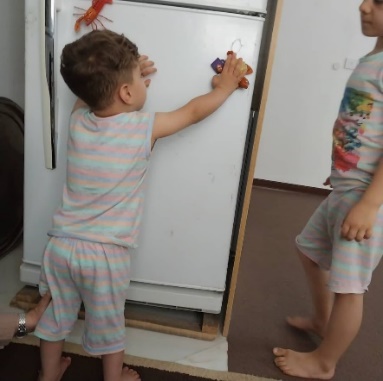

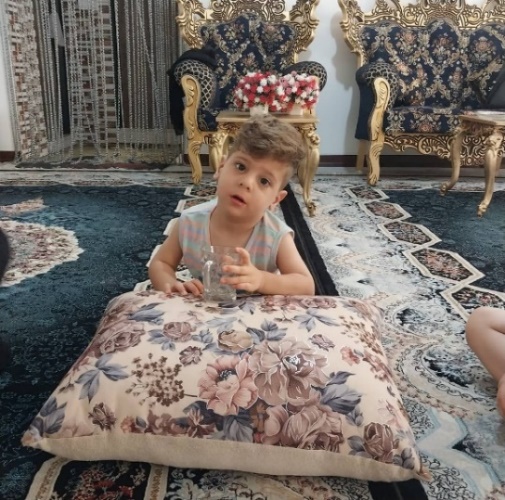

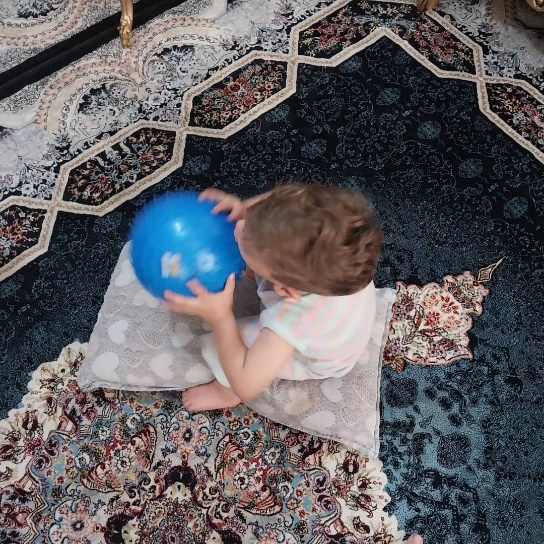

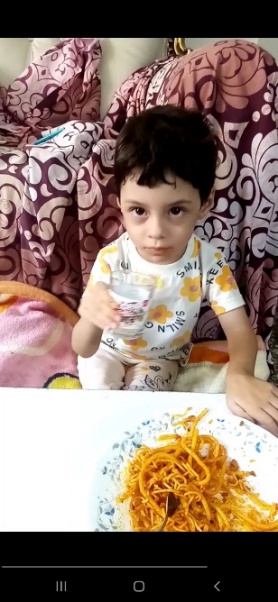

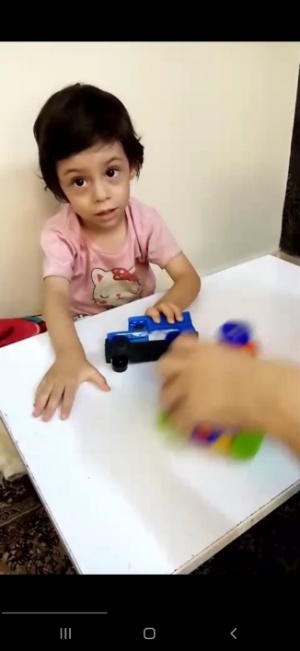


**1**

**2**

**3**

**4**

**5**

**6**

**7**

**8**


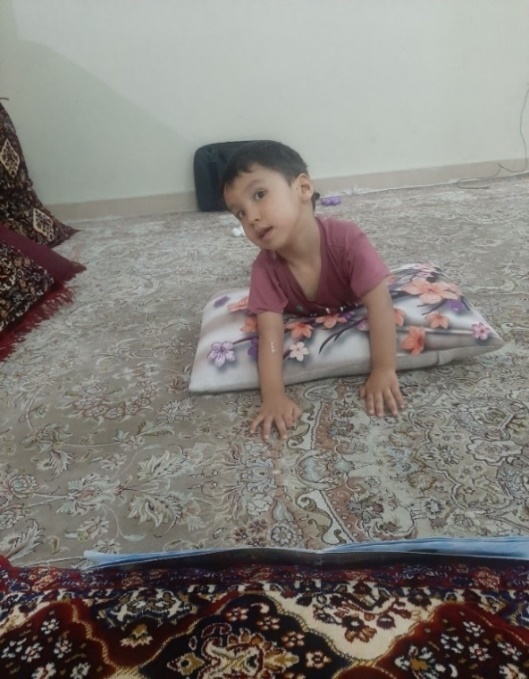

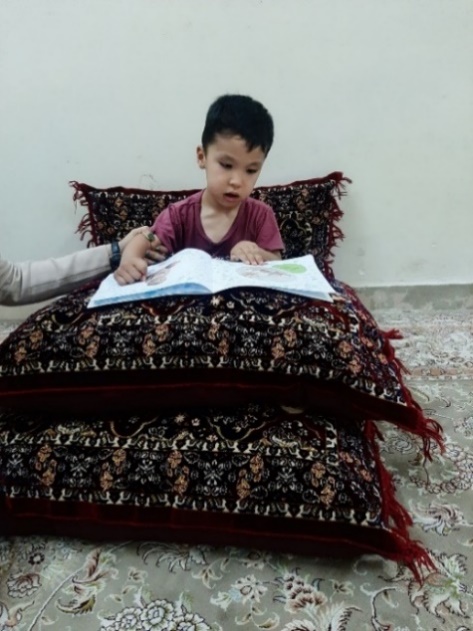

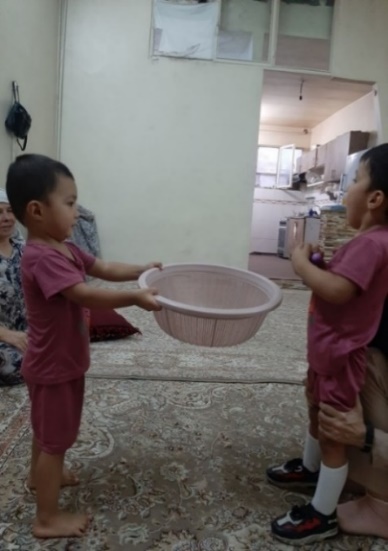

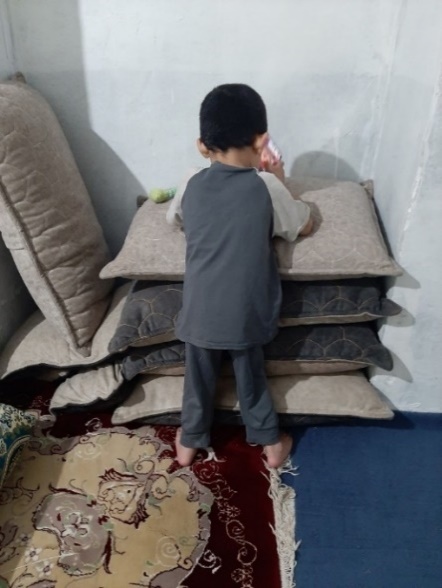


1. Participant 1: Academic tasks, short sit to straighten the trunk, maintain 90-degree of the ankles, prevent the hanging of foot (GMFCS4)

2. Participant 1: Play (cooking), kneeling and hip extension, bearing weight on the knees (GMFCS4)

3. Participant 1: Play (cooking), Standing and using hands without trunk support (GMFCS4)

4. Participant 2: Manual games, standing and using hands with left knee support (GMFCS3)

5. Participant 2: Eating, sitting with a hip abduction to prevent w-sitting, using a pillow as a table (GMFCS 3)

6. Participant 2: Play, Short sit with thighs separated to prevent w-sitting (GMFCS3)

7. Participant 3: Eating, Short sit and using the writing desk as a dining table, leaning on the left wrist to support the trunk (GMFCS4)

8. Participant 3: Play. Short Sit, using a writing desk and leaning on the right hand to support the trunk (GMFCS4)

9. Participant 4: Watching TV, prone on wrists to facilitate head control and weight bearing on (GMFCS4)

10. Pparticipant 4: Cognitive games, sitting on a pillow, using pillows as a table, leaning on elbows to support the trunk (GMFCS4)

11. Pparticipant 4: Playing with peers, standing with support on knees and hips (GMFCS4)

12. Participant 5: Play, prone standing on Stacked pillow (GMFCS3)

**12**

**11**

**9**

**10**

**\\**

Abbreviation**:** GMFCS= Gross Motor Function Classification System

Note: From left to right in each line: Image number. participant number: occupation, suggested position (participant GMFCS level).

**SUPPLEMENTARY 2:** Examples of Household Items Used for Occupational Positioning

| **No.** | **Household Item** | **Functional Use and Application** |
| --- | --- | --- |
| 1 | Adult Dining Chair | • Kneeling in front of the chair to use the seat as a work surface for correct trunk alignment (**Participants 1, 2**).  • Sitting on a cushion/stool or standing in front of the chair to use it as a table (**Participant 2**).  • Standing behind the chair, holding the backrest for speech games and watching TV (**Participants 1, 2**).  *Chair stability was ensured by placing it* in front of *a wall or having a caregiver hold it.* |
| 2 | In Front of the Sofa | • Kneeling and using the sofa seat as a desk for hand-based games or educational activities (**Participants 1, 2, 3**).  • Short-sit on a stool/cushion to use the seat as a table for play or eating (**Participants 1, 2, 3**).  • Walking along the sofa by leaning on wrists during mobile play (**Participant 2**).  *The sofa seat provided a wide and stable surface under supervision.* |
| 3 | Sofa Cushions and Pillows | • Use sofa cushion under hip during short sit to perform daily tasks (**Participant 1**). •used One cushion (ring/long-sit), two (short-sit) as a desk in front of the child for sitting occupations, or 2–5 stacked as a table for kneeling or standing occupations (**All participants**).  • Under feet when sitting on a chair to maintain 90° ankle posture and prevent plantar flexion (**All participants**).  • A sofa cushion is used to raise the child while standing in front of the kitchen counter to reduce trunk support and help the child access the kitchen counter (**Participant 1**).  • One or two pillows under hips for short-sit (**Participants 4, 5**).  • Standing on the back of the wall and placing pillows vertically on both sides to support the standing position (**Participant 4**).  *Caregiver support is required to stabilize stacked cushions used as a table.* |
| 4 | Sofa Armrests | • Standing in front of the armrests of adjacent sofas to create a support surface, the Sofa armrest provides a small work surface (**Participants 1, 3**).  • Standing with back to the wall between two sofa armrests (**Participants 1, 2, 3**).  • Standing between two sofa armrests, one in front and one behind, with the front armrest acting as a table (**Participant 3**).  *Recommended for short-term tasks with caregiver supervision.* |
| 5 | Kitchen Counter | • Standing in front of the kitchen counter and using it as a large, stable surface for play (**Participant 1**).  *A cushion was placed under the feet to reduce trunk control and increase surface access.* |
| 6 | Round Pillow | • Used to maintain hip abduction during short sit, particularly while seated for activities such as TV watching (**All participants**). |
| 7 | Writing Desk | • Standing with back supported by a wall and wrists resting on the front-positioned desk (**Participants 1, 3**). |
| 8 | Wall / Corner Wall | • Used for back support in standing or during long-sit position to support the trunk (**All participants**). |
| 9 | Window Ledge | • Kneel walking by leaning on elbows or supporting wrists on the ledge (**Participant 5**). |
| 10 | Child’s Ride-On Toy | • Kneeling while holding and pushing the toy for mobility during occupational tasks (**Participant 2**). |
